# Benefits of Dental Scaling and Polishing in Adults: A Rapid Review and Evidence Synthesis

**DOI:** 10.1177/23800844241271684

**Published:** 2024-10-09

**Authors:** D.C. Matthews, H. Al-Waeli

**Affiliations:** 1 Faculty of Dentistry, Dalhousie University, Halifax Nova Scotia, Canada

**Keywords:** periodontal diseases, access to care, dental care, oral hygiene, cost, health policy

## Abstract

**Background:**

This rapid review assessed evidence to inform policy on the clinical effectiveness and optimal frequency of dental scaling and polishing (S&P) for adults, including those with low incomes eligible for the Canadian Dental Care Plan.

**Methods:**

A rapid review was conducted according to Cochrane Recommendations for Rapid Reviews. Populations included all adults, adults with periodontitis, and those with inequitable access to dental care. Primary outcomes included gingival inflammation, probing depths, and tooth loss. Secondary outcomes included oral health–related quality of life and economic impact. Four databases were searched for randomized clinical trials, systematic reviews, cohort studies, and practice guidelines. Risk of bias was evaluated using Cochrane Risk of Bias, Newcastle-Ottawa, ROBIS, and AGREE II tools. A qualitative synthesis was planned.

**Results:**

In total, 3,181 references were retrieved: 4 applied to “all adults” and 4 to those with periodontitis. All reports had low risk of bias. One systematic review and one multicenter trial of adults with regular dental care found no clinical benefit regardless of S&P interval; however, patients valued and were willing to pay for regular scaling. One claims-based study reported regular S&P reduced tooth loss, and 2 clinical practice guidelines found a reduced risk of future attachment and tooth loss, lower overall health care costs for diabetes, and reduced costs for and incidence of acute myocardial infarction in those with regular S&P. There were no studies of underserved populations.

**Conclusions:**

For adults with no or early periodontal disease and regular access to dental care, routine S&P may have little clinical benefit but reduces tooth loss and some health care expenses. In patients with periodontitis, scaling intervals tailored to individual risk profile and periodontal status can maintain health. There is no evidence on the impact of routine S&P on patients with barriers accessing care.

**Knowledge Transfer Statement:**

In terms of the benefits of routine scaling and polishing in adults, this rapid review found mixed evidence with a high level of certainty due to minimal risk of bias in the appraised studies for “regular dental attenders” and those with a diagnosis of periodontal diseases. Tailored intervals for dental scaling are beneficial for those diagnosed with periodontitis but may not provide the clinical benefits previously expected for adults at low risk. There is no evidence that dental polishing is effective. No evidence was found to support recommendations about the clinical effectiveness of scaling or the most appropriate recall intervals for scaling for low-income Canadians eligible for dental services under the new Canadian Dental Care Plan.

## Introduction

*Periodontitis* is a broad term representing a cluster of diseases that result in inflammatory responses and chronic destruction of the periodontium—ranging from reversible gingivitis to irreversible loss of soft tissue and alveolar bone (periodontitis). Depending on the level of attachment loss that occurs, patients may experience impaired chewing and even tooth loss—affecting an individual’s nutrition and overall quality of life.

The economic burden of periodontal diseases is considerable. As such, periodontitis is considered a source of social inequality, not the least of which is related to the significant costs involved in replacing missing teeth resulting from periodontitis (Tonetti et al. 2017). Furthermore, as a noncommunicable disease (NCD), periodontitis shares social determinants and risk factors with other major NCDs (cardiovascular diseases, diabetes, cancer, and respiratory disease) that cause two-thirds of all deaths (Sanz et al. 2018; Sanz, Marco Del Castillo, et al. 2020) and significantly affect national health care budgets. The global cost of lost productivity from severe periodontitis alone has been estimated to be 54 billion USD/year (Tonetti et al. 2017).

In most higher-income countries, it is the accepted norm for most people to attend dental check-ups at 6-mo intervals to maintain gingival and periodontal health. These appointments usually include a “basket of services” that includes examination for a range of head and neck and oral conditions, professional mechanical plaque removal (PMPR) or “scale and polish” (S&P), and oral hygiene instruction (OHI) or advice (OHA). For those with a history of periodontitis, who are at a higher risk of future attachment loss, there is substantial evidence to support PMRP every 3 to 12 mo (referred to as periodontal maintenance or supportive periodontal therapy [SPT]) depending on the disease activity, extent, and severity (Jepsen et al. 2017; Trombelli et al. 2020).

Recently, the benefits of regular 6-mo S&P appointments have been challenged. A 2018 Cochrane Review by Lamont et al. (2018) reported little or no difference in gingival inflammation for regular S&P treatments compared with no treatment or between 6- and 12-mo intervals. The data from the included studies are from “regular dental attenders” who tend to be at low risk of future periodontal attachment loss for a variety of care. They are more likely to belong to higher socioeconomic groups, be healthier, and face fewer challenges accessing regular dental care than those who experience a variety of barriers to dental care (Thompson et al. 2014).

In fact, periodontal diseases have been shown to disproportionately affect those with inequitable access to dental care. Canadians from lower-income families have almost 2 times worse oral health outcomes than higher-income Canadians (Health Canada 2010). Two-thirds of those in long-term care facilities have been found to have periodontal disease, compared to half of those living independently in the community (McNally et al. 2014). However, these populations are also the least likely to be able to afford dental care. A 2022 survey by Statistics Canada reported 48% of families with a net income under CDN $70,000 and 23% of those with a net income less than CDN $90,000 have no private or government-paid insurance to cover dental expenses. Similarly, 58% of adults aged 65 y or older report no insurance coverage. Nearly 85% of recent immigrants and refugees reported moderate to severe periodontitis, none of whom had dental insurance (Ghiabi et al. 2014).

Canada lags behind other Organisation for Economic Co-operation and Development (OECD) countries in terms of public funding for dental care for the underserved, ranking in the lower quartile (OECD 2023). In 2022, the Canadian parliament approved a bill for the implementation of the Canadian Dental Care Plan (CDCP) and in 2023 pledged $13 billion over 5 y to ensure families without dental insurance and whose income is less than $90,000 a year would have access to dental care. As this plan is being rolled out, Health Canada contracted an evidence review to help determine the cost-effectiveness of various routine dental procedures, including “routine S&P” for the Canadian population.

Updating Lamont’s review (Lamont et al. 2018), the purpose of this study is to provide an appraisal and synthesis of the best evidence from 2018 to 2023 and aid policymakers in the determination of the types of services and optimal frequency of those services for the improvement and maintenance of gingival and periodontal health of Canadian adults, including older adults and underserved or vulnerable populations.

## Methods

A “rapid review” (King et al. 2022) and evidence synthesis were conducted to determine the types of services and optimal frequency of those services for the improvement and maintenance of gingival and periodontal health of the Canadian population. A rapid review is a modification of the systematic review process designed to produce information that can be used to inform policy in a shorter time frame. Although methods vary, the process is streamlined to focus on the needs of the end user, and evidence synthesis does not necessarily include meta-analysis. Recommendations from the Cochrane Rapid Reviews Methods Group (Garritty et al. 2021) were followed. This includes setting the research question and eligibility criteria, searching and study selection, data extraction, risk of bias assessment, and evidence synthesis.

### Setting the Research Question

The review was commissioned by Health Canada’s Dental Care Task Force in preparation for the rollout of the CDCP. The protocol was submitted to the stakeholder prior to conducting the review, and the authors consulted with the stakeholders throughout the process. The objectives of the review were 2-fold:

1. To evaluate the effectiveness of routine scaling and polishing compared to no scaling and polishing for improving periodontal outcomes in adults with periodontal health, adults with periodontitis, and adults with limited access to regular care
2. To evaluate the effectiveness of routine scaling and polishing at different recall intervals for periodontal health in adults

Following Preferred Reporting Items for Systematic Reviews and Meta-Analyses (PRISMA) guidelines for Systematic Review Protocols (Page et al. 2021), we established the research questions using the PICOS format (population, intervention, comparator, outcome, study design).

### Criteria for Considering Studies for This Review

#### Type of studies

All clinical practice guidelines, systematic reviews, randomized controlled trials, controlled clinical trials, and cohort studies were included. When systematic reviews were identified, primary studies were only included if they were not included in systematic reviews. The minimum duration of clinical trials was 1 y. Studies were included only if changes in periodontal outcomes were reported; thus, case control studies were excluded.

#### Type of participants

We included studies involving dentate adults (no upper age limit) with periodontal health and those with a history of periodontal disease. We also included studies that identified dentate adults with limited access to regular dental care (adults living in long-term residential care, living in rural remote areas, those with low incomes, and without dental insurance).

#### Types of interventions

Included were studies where “routine scale and polish” (S&P or PMRP) was provided by a dental care professional (dentist, dental therapist, or dental hygienist) with or without oral hygiene instructions). S&P is defined as removal of bacterial plaque, mineralized plaque deposits (calculus), debris, and staining from the crowns and roots of teeth through rubber cup polishing or air polishing techniques, with or without the use of mechanical (sonic, ultrasonic, or piezo) or hand (scalers, curettes) instruments. Also included were studies in which patients with a history of treated periodontitis were enrolled in SPT.

For each of the populations of interest (periodontally healthy adults, adults with periodontitis, and those with limited access to dental care), we compared routine scaling and/or polishing (or SPT in the case of patients with a history of periodontitis) planned at regular intervals (e.g., every 3, 6, or 12 mo) compared to no scheduled treatment and compared to different recall intervals (e.g., 12 or 24 mo).

#### Types of outcome measures

We included trials reporting clinical status, participant-centered outcomes, and cost outcomes. Primary outcomes include tooth loss and gingival and periodontal health as determined by measures of inflammation (bleeding on probing, gingival indices, gingival bleeding). For patients previously treated for periodontitis, changes in probing pocket depth were included.

Secondary outcomes include costs of S&P and impact on oral health–related quality of life (OHQoL).

### Search Methods for Identification of Studies

A health information specialist conducted a search of registries and databases based on that of Lamont et al. (2018), updating the search to include the period January 2018 to December 2023 and following the inclusion criteria listed above. There were no language or publication status restrictions.

Four databases were searched: Cochrane Oral Health Trials Register, Cochrane Central Register of Controlled Trials (CENTRAL), MEDLINE Ovid, and Elsevier Embase (Appendix 1). Gray literature was not searched. Reference lists of related articles for relevant reports were reviewed and the full text of those reports that appeared to meet the eligibility criteria retrieved. Ongoing studies or protocols were not included.

### Data Collection and Evidence Synthesis

#### Selection of studies

All records were screened in duplicate by the 2 authors (D.M. and H.A.-W.). Duplicate studies were removed or merged. Both authors performed the primary search by screening independently the titles and abstracts.

The full report for all studies appearing to meet the inclusion criteria or in instances when there was insufficient information from the title or abstract to make a clear decision was obtained. Instances of disagreement in the study selection process were resolved by mutual discussion between review team members.

#### Data extraction and synthesis

One author (D.M.) extracted data, recording study design, location(s), number of participants recruited and evaluated, intervention, comparator, and primary and secondary outcomes. A second author (H.A.-W.) independently audited the included studies for their suitability for inclusion and the interpretation of their findings.

A narrative review (qualitative synthesis) of the evidence was planned. The evidence was synthesized by population type. Due to the significant heterogeneity in the methods and outcomes used across the included studies, quantitative syntheses, sensitivity analyses, subgroup analyses, and publication bias assessment were deemed inappropriate (Higgins 2019).

### Risk of Bias Assessment of Included Studies

All included studies were assessed for risk of bias using the Cochrane Risk of Bias 2 tool for randomized trials (University of Bristol 2021), the Newcastle-Ottawa Scale (NOS) (Ottawa Hospital Research Institute 2021) for cohort studies, and the Risk of Bias for Interventional Studies (ROBIS 2023) tool for systematic reviews. The methodologic rigor and transparency of guideline development for Clinical Practice Guidelines was evaluated using the AGREE II methodology (Brouwers et al. 2010). The risks of bias and quality of guideline development were incorporated into the final recommendations.

## Results

### Search Results

**Figure 1.**
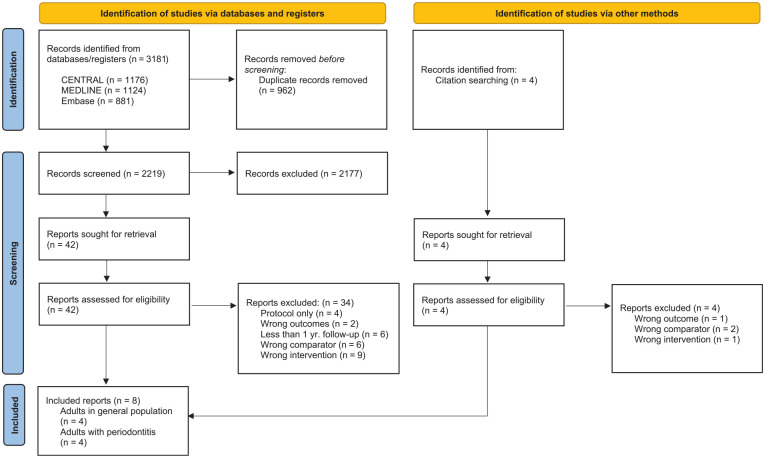
Preferred Reporting Items for Systematic Reviews and Meta-Analyses (PRISMA) flow diagram. Selection of included reports.

The results of the search are summarized in Figure 1.

The electronic searches yielded 3,181 references. Following removal of duplicate records, this was reduced to 2,219. Two review authors screened the titles and abstracts against the inclusion criteria, independently and in duplicate, discarding 2,177 references in the process. We obtained full text copies of 42 references and identified 4 additional reports not previously retrieved. Of the 46 records assessed for eligibility, 38 did not meet the inclusion criteria. Eight records met all inclusion criteria—4 for the population of “all adults” and 4 for those “adults with a history of (having been treated for) periodontitis.”

No studies met the inclusion criteria for adults with limited access to dental care (i.e., living in long-term care, those with low incomes with or without third-party dental insurance).

No studies were retrieved comparing “scaling” to “polishing” or “polishing” to no treatment in any of the populations of interest.

Table 1 lists the details of the characteristics of included studies.

**Table 1.** Characteristics of Included Reports. Adults from General Dental Practices.

Ramsay et al. (2018) and Clarkson et al. (2021)	
Methods	Design: Multicenter RCT Location: UK Setting: Primary (general) dental care offices Number of locations: 63 practices
Participants	Dentate or partially dentate adults receiving regular dental care. Patients with moderate to advanced periodontitis (clinical probing depth of >6 mm and/or furcation involvements or attachment loss of ≥7 mm) and those with an uncontrolled chronic medical condition (e.g., diabetes mellitus, immunocompromised) were *excluded*. Number of participants: 1,877 randomized; 1,327 analyzed
Interventions	Scale and polish (PI): Included the removal of plaque and calculus from the crown and root surfaces using manual or ultrasonic scalers and appropriate management of plaque-retentive factors but no adjunctive subgingival (e.g., local antibiotic) therapy. **Group 1:** Scale and polish at 6 mo **Group 2:** Scale and polish at 12 mo **Group 3:** No scale and polish **Time frame:** 3 y
Outcomes	**Bleeding on probing:** No statistical or clinical differences between 0 versus 6 mo (difference 0.87%; 95% CI, –1.6% to 3.3%; *P* = 0.481). There was a <1% difference in the average number of sites with gingival bleeding between the randomized groups. No statistical or clinical differences between 6 and 12 mo (difference 0.11%; 95% CI, –2.3% to 2.5%; *P* = 0.929). **Clinical probing depth:** No statistical or clinical differences between 0 versus 6 mo or between 6 and 12 mo. **Cost-benefit analysis:** They reported on the incremental net benefit relative to standard care (routine OH advice with 6-monthly PI). A within-trial cost-benefit analysis found the group randomized to receive no PI had the lowest average costs to the NHS. Accounting for health and nonhealth costs and assuming the aim is to maximize welfare from a fixed NHS budget, 6-monthly PI with personalized OH advice had the largest incremental net benefit compared to standard care (difference £48; 95% CI, £22 to £74). **WTP:** In a separate substudy of a nationally representative online sample of the UK general population, a DCE was used to estimate WTP. The general population highly valued both PI and personalized OHA even when controlling for bleeding gums and aesthetics. The 6-monthly PI with personalized OHA had the greatest benefit (mean difference vs. standard care £61.67; 95% CI, £40.19 to £83.14). A 12-monthly PI with personalized OHA also had positive, but not significant, incremental benefits.
Notes	A clinically important difference in bleeding on probing was set at 7.5%.
Kao et al. (2021)	
Methods	**Design:** Retrospective cohort of health claims data from the LHID **Location:** Taiwan
Participants	Adults aged 50 to 64 y Number of records: 14,328
Interventions	**Group 1:** Regular scaling (once or twice a year) **Group 2:** No scaling **Time frame:** 2000–2013
Outcomes	**Risk of AMI:** Incidence rate of AMI in the group who received no tooth scaling was significantly higher (3.5%), compared to those who had scaling (1.9%; hazard ratio = 0.543; 95% CI, 0.441–0.670). **Costs:** Lump-sum annual expenditure of tooth scaling plus treatment for AMI therapies was lower than those who did not have tooth scaling (US $265.76 vs. US $292.47).
Notes	In Taiwan, dental scaling is provided for each beneficiary once every 6 mo. Opportunity costs were not included.
Lamont et al. (2018)	
Methods	**Design:** Cochrane systematic review of RCTs
Participants	Adults (aged 18–92 y) without severe periodontitis who were regular attenders at UK general dental practices, followed up for 24 to 36 mo **Number of RCTs:** 4 **Number of participants:** 1,086
Interventions	**Group 1:** Routine S&P 6 mo **Group 2:** Routine S&P 12 mo **Group 3:** No scale and polish
Outcomes	**Gingivitis:** (High certainty of evidence) Little to no difference between groups *6 mo vs. no S&P*: SMD = −0.01 (95% CI, –0.13 to 0.11); 2 trials, 1,087 participants; *I*^2^ = 0% *12 mo vs. no S&P*: SMD = −0.04 (95% CI, –0.16 to 0.08); 2 trials, 1,091 participants; *I*^2^ = 0% *6 mo vs. 12 mo*: SMD = −0.04 (95% CI, –0.16 to 0.08); *I*^2^ = 0% **Quality of life (OHIP-14):** (High certainty of evidence) Little to no difference between groups *6 mo vs. no S&P*: MD = −.030 (95% CI, –1.24 to 0.64) *12 mo vs. no S&P*: MD = 0.10 (95% CI, –0.83 to 1.03) **Patient perception of oral cleanliness:** (Low certainty of evidence) Participants receiving 6-monthly and 12-monthly S&P treatments reported higher levels of oral cleanliness compared to no scheduled S&P. *6 mo vs. no S&P*: RR = 1.83 (95% CI, 1.28–2.63) *12 mo vs. no S&P*: RR = 1.65 (95% CI, 1.13–2.40) **Cost:** (Very low certainty of evidence): 1 trial, 554 participants From an NHS perspective only, there were no differences in terms of costs for any intervals compared. *6 mo vs. no S&P*: MD = GBP 00.52 (95% CI, –£18.10 to £19.14) *12 mo vs. no S&P*: MD = GBP 8.14 more (95% CI, £13.76 less to 30.04 more) *6 mo vs. 12 mo*: MD = GBP 7.62 less (95% CI, £28.39 less to £13.15 more)
Notes	SMD <0.40 represents a small difference; 0.40 to 0.70, a moderate difference; >0.70, a large difference. OHIP-14: A difference <2.0 points is unlikely to be perceived as important by patients.
Lee et al. (2019)	
Methods	**Design:** Prospective study of Korean Genome and Epidemiology Study on Atherosclerosis Risk of Rural Areas in the Korean General Population
Participants	Dentate adults aged 40 to 75 y **Number of participants:** 557
Interventions	**Group 1:** Regular dental scaling **Group 2:** Irregular or no dental scaling **Time frame:** 3 y
Outcomes	**Tooth loss:** 81 participants (14.5%) had regular dental scaling and 291 (52.2%) did not receive any dental scaling at baseline or over the 3 y. The incidence of tooth loss was 1.87 (CI, 1.03–3.38) higher in participants who did not receive scaling during the 3-y period than in those who received scaling regularly, even after adjusting for confounding factors (education, age, marital status).
Notes	

**Table:** Adults Treated for Periodontitis.

Smits et al. (2020)	
Methods	**Design:** Retrospective cohort of claims data **Location:** Netherlands
Participants	Adults aged 18 to 100 y Number of participants: (*n* = 934,704, of whom 43,678 claimed at least 1 diabetes-related health care reimbursement; 8,188 with periodontal treatment, 33,410 without)
Interventions	**Group 1:** “Intermediate” periodontal care reimbursed (i.e., initial therapy and SPT, but no periodontal surgery) **Group 2:** No periodontal care reimbursed **Time frame:** 7 y
Outcomes	**Costs:** Based on fixed-effects regression analysis, intermediate periodontal treatment resulted in a reduction in expenses per quarter per individual of €8.04 (95% CI, –€12.80 to –€3.28; *P* < 0.001)
Notes	Dutch Periodontal Screening Index (validated instrument) used to screen for individuals with no, mild, and severe periodontitis. Individuals with no periodontitis are not reimbursed for periodontal care. A panel regression fixed-effects model was used to control for unobserved (time-invariant) individual characteristics. Several sensitivity analysis conducted.
Manresa et al. (2018)	
Methods	**Design:** Cochrane Systematic Review
Participants	Adults aged 18 to 85 y with treated periodontitis enrolled in periodontal SPT for at least 6 mo **Number of participants:** No studies found
Interventions	**Group 1:** Regular SPT **Group 2:** Monitoring only **Group 3:** Different recall intervals
Outcomes	**Signs of inflammation:** No studies found for comparison groups
Notes	This review examined other comparison groups not related to this evidence synthesis (i.e., adjuncts to scaling; specialty vs. general dental care). These were not included in this evidence synthesis.

AMI, acute myocardial infarction; CI, confidence interval; DCE, discrete choice experiment; GBP, British pounds; LHIS, Longitudinal Health Insurance Database; MD, mean difference; NHS, National Health Service; OH, oral health; OHA, oral hygiene advice; PI, periodontal instrumentation; RCT, randomized controlled trial; RR, relative risk; S&P, scale and polish; SMD, standardized mean difference; WTP, willingness to pay.

### Summary of Evidence

#### Primary outcome: clinical health

Clinical outcomes included measures of gingival inflammation, probing pocket depths, and tooth loss.

### Population: All Adults

In 2021, Clarkson et al. reported on a multicenter, multilevel cluster randomized factorial open trial with blinded outcome evaluation. This report was based on Ramsay et al.’s (2018) study for the UK National Institute for Health Research Health and Technology Assessment IQuaD (Improving the Quality of Dentistry). The trial involved adults who were “regular dental attenders” with no or early signs of periodontitis. They were randomized within 63 UK general dental practices to receive S&P at 6- or 12-mo intervals or not at all. All participants were encouraged to attend for a routine examination every 12 mo. After 3 y, there were no statistically significant clinical benefits from 6-monthly or 12-monthly scale and polish over no S&P.

In an update of a 2013 Cochrane review, Lamont et al. (2018) included studies of dentate adults without severe periodontitis who attended UK general dental practices for routine S&P. Two randomized controlled trials (RCTs) were included (*n* = 1,087), both of which were deemed to be at low risk of bias. In adults without severe periodontitis who regularly access routine dental care, routine S&P compared with no scheduled scale and polish treatments made little or no difference to gingivitis or probing depths over 2- to 3-y follow-up. Not surprisingly, calculus levels were reduced compared to those who did not have routine S&P, but the clinical implications of this finding were unclear since there was no difference in inflammation.

In terms of tooth loss, 1 cohort study met the inclusion criteria. Lee et al. (2019) conducted a 3-y prospective cohort based on the Korean Genome and Epidemiology Study on Atherosclerosis Risk of Rural Areas in the Korean General Population. The population included dentate adults aged 40 to 75 y (*n* = 557). The incidence of tooth loss was higher in participants who did not have their teeth scaled during the 3-y period than in those who received scaling regularly even after adjusting for socioeconomic factors.

### Population: Adults with a History of Periodontal Disease

A Cochrane systematic review by Manresa et al. (2018) evaluated the effect of SPT in adults with treated periodontitis. No eligible RCTs were retrieved that evaluated SPT versus monitoring only, SPT provided at different time intervals, or the effects of scaling using different approaches or technologies.

Two S3 Clinical Practice Guidelines (Sanz, Herrera, et al. 2020; Herrera et al. 2022) were recently published that addressed questions around the diagnosis, management, and long-term disease prevention of adults with periodontitis and the impact of patient-reported outcomes, including cost, quality of life, and impact on systematic health conditions. They were developed under the auspices of the European Federation of Periodontology (EFP) following the methodologic guidelines of the Scientific Medical Societies in Germany (AWMF) (Nothacker et al. 2014). A broad group of international stakeholders participated in the process. S3 guidelines and recommendations are the highest level of guidelines; they are evidence based—following AGREE II methodology (Brouwers et al. 2010), developed using a formal consensus-building process, and graded on the strength of the evidence as determined by the Grading of Recommendations Assessment, Development, and Evaluation (GRADE) criteria (www.gradeworkinggroup.org) (Andrews et al. 2013) (Table 2).

**Table 2.** Strength of Recommendations: Grading Scheme for Clinical Practice Guidelines (Andrews et al. 2013; Nothacker et al. 2014).

Grade of Recommendation	Description	Syntax
**A**	Strong recommendation	We recommend/recommend not to
**B**	Recommendation	We suggest/suggest not to
**O**	Open recommendation	May be considered

Recommendations from the guidelines are detailed in Table 3. Strong recommendations are based on grade A level evidence and strong or unanimous consensus of experts. In summary, it is strongly recommended that PMPR and control of plaque-retentive factors be included in the first step of periodontal therapy for patients diagnosed with early to advanced (i.e., stage I to IV; Tonetti et al. 2018) periodontitis. In patients who have completed active (i.e., nonsurgical and/or surgical) periodontal therapy, it is strongly recommended that, to reduce the risk of tooth loss and prevent disease recurrence, regular professional SPT be provided and adhered to. It is strongly recommended that initially, SPT be scheduled at 3-mo intervals. Thereafter, the schedule should be tailored according to patients’ risk profile and periodontal conditions, with high-risk individuals benefiting from 3-monthly SPT and lower-risk patients remaining largely stable with a frequency of 6 to 12 mo.

**Table 3.** Summary of Clinical Guideline Recommendations for Adults with Periodontitis (Sanz et al. 2018; Sanz, Herrera, et al. 2020; Herrera et al. 2022).

Question	Recommendation	Grade of Recommendation^a^/ Strength of Consensus
What is the efficacy of supragingival PMPR and control of retentive factors in periodontitis therapy?	**Strongly recommend** supragingival PMPR and control of retentive factors, as part of the first step of therapy.	Grade A Expert consensus-based recommendation based on substantial indirect evidence and unanimous consensus.
Does regular SPT reduce tooth loss or prevent disease recurrence in the long term?	**Strongly recommend** provision of and adherence to regular professionally administered SPT to reduce tooth loss in the long term (≥5 y) and minimize loss of periodontal attachment.	Grade A Evidence-based recommendation based on SR of 17 prospective cohorts at low risk of bias and 1 at moderate risk of bias (tooth loss), 7 prospective cohorts at low risk of bias (clinical attachment loss ≥2 mm), and unanimous consensus.
At what intervals should SPT be scheduled?	**Strongly recommend** that supportive periodontal care visits be scheduled at intervals of 3 to a maximum of 12 mo and ought to be tailored according to the patient’s risk profile and periodontal conditions after active therapy.	Grade A Expert consensus-based recommendation based on 4 SRs and strong consensus.
Should recall intervals for SPT be guided by patients’ risk status?	**Strongly recommend** recall intervals for SPT should be guided by patients’ risk profile as determined by individual risk factors (e.g., smoking, hyperglycemia) and disease-associated clinical measures (such as pocket depths and bleeding on probing), with high-risk individuals benefiting from 3-monthly SPT and lower-risk patients remaining largely stable with a frequency of 6 to 12 mo.	Grade A Expert consensus-based recommendation based on 1 cohort study at low risk of bias, indirect evidence from 6 SRs, and unanimous consensus.
What is the value of PMPR as part of SPT?	**Recommend** performing routine PMPR to limit the rate of tooth loss and provide periodontal stability/improvement, as part of a supportive periodontal care program.	Grade A Expert consensus-based recommendation based on 1 SR and strong consensus.
What is the value of oral hygiene instructions for patients as part of SPT?	**Strongly recommend** repeated individually tailored instructions in mechanical oral hygiene, including interdental cleaning, in order to control inflammation and avoid potential damage for patients in periodontal SPT.	Grade A Evidence-based recommendation based on 1 SR and unanimous consensus
Are there disadvantages to regular long-term SPT (e.g., increased gingival recession/clinical attachment loss)?	There is **inadequate evidence** of clinical disadvantages to regular long-term SPT, such as gingival recession/clinical attachment loss; however, the possibility of these side effects cannot be excluded based on the evidence reviewed. Patients should be advised of this as part of their informed consent.	Grade O Evidence-based statement Based on 3 prospective cohorts at low risk of bias and strong consensus. Additional evidence needed.
Is long-term SPT cost-effective when considering direct and indirect costs?	**Suggest** that regular long-term SPT in specialist practice may result in greater periodontal stability and tooth survival when compared with SPT in general practice. It is unknown if provision of care in a specialty office is cost-effective when considering direct and indirect costs.	Grade O Expert consensus derived statement based on 1 SR and unanimous consensus. Additional evidence needed.
Does long-term SPT affect patient-reported outcome measures (OHRQoL, masticatory function, aesthetics)?	There is **inadequate evidence** to determine if long-term SPT impacts patient-reported outcomes.	Grade O Evidence-based statement based on indirect evidence from 3 prospective cohorts and unanimous consensus. Additional evidence needed.

OHRQoL, oral health–related quality of life; PMPR, professional mechanical plaque removal; SPT, supportive periodontal therapy; SR, systematic review.

a See Table 2.

The risk profile should be determined by individual risk factors (e.g., smoking, hyperglycemia, personal plaque control) and disease-associated clinical measures (such as pocket depths and bleeding on probing). It is strongly recommended that SPT visits include delivery of care (including PMPR and treatment of sites with active or recurrent disease) by oral health care professionals, under the supervision of a suitably trained general dentist or specialist as appropriate to the case complexity, and individualized oral hygiene instructions, tailored to each patient’s needs.

Regular long-term SPT in a specialist practice may result in greater periodontal stability and tooth survival when compared with SPT in general practice, but it is unknown if provision of care in a specialty office is cost-effective when considering direct and indirect costs.

The group found no evidence of clinical disadvantages to regular long-term SPT, such as gingival recession or clinical attachment loss, and recommends additional research in this area.

While a number of recommendations in these studies determined that “periodontal treatment” may have a positive impact on a variety of systemic health conditions, they did not specify the effect of routine SPT.

### Secondary Outcomes

#### Quality of life

Lamont et al. (2018) reported that one of the studies retrieved in their systematic review included self-report data from participants. Those who had 6- or 12-mo scaling reported feeling their teeth were cleaner than those who had no treatment. The difference was small and based on a very low certainty of evidence. There was no difference between groups in terms of other quality-of-life outcomes. Similarly, among regular dental attenders at low risk of periodontitis, Ramsey et al. (2018) reported no difference in oral health–related quality of life between intervention arms in any comparison.

#### Economic impact

In the study by Ramsey et al. (2018), a within-trial cost-benefit analysis assessed the costs and benefits (in monetary terms) of each policy compared with standard care (routine OHA with 6-monthly scaling). There were no significant differences in National Health Service (NHS) dental costs.

This study included a discrete choice experiment, administered to a nationally representative online sample of the UK general population to estimate individuals’ “willingness to pay” out of pocket for care. The results showed that the general population valued both scaling and personalized OHA, with greater financial value placed on scaling than on OHA.

In the systematic review by Lamont et al. (2018), the level of evidence on the impact of costs of routine S&P treatments to the NHS was uncertain based on very low-quality evidence. The patient perspective on costs determined by WTP (as considered in the Ramsey et al. [2018] study) was not included in their analysis.

Many chronic health conditions, including diabetes, are more commonly found among those in lower socioeconomic classes (GBD 2016). The prevalence of periodontitis is higher in patients with diabetes than those without diabetes. Periodontitis is often referred to as the sixth complication of diabetes mellitus, with evidence of a strong bidirectional relationship between the 2 conditions. Those individuals diagnosed with diabetes who also have periodontitis are more likely to have severe periodontitis, exhibit poorer metabolic control, and are more likely to experience diabetic complications (Sanz et al. 2018).

To examine the impact of the treatment of periodontitis on diabetes-related health care costs, Smits et al. (2020) conducted a retrospective analysis of 6-y claims data in the Netherlands (*n* = 937,704 patients, of whom 43,678 claimed diabetes-related health care reimbursement). All reimbursement costs and number of claims for periodontal treatment and diabetes-related health care were extracted per quarter of a year. They reported a significant reduction in total diabetes-related health care costs for patients who received periodontal treatment compared with no periodontal treatment (approximately €9 to €38 per patient per year). This included initial nonsurgical periodontal therapy and SPT and was mainly attributable to a reduction in health care costs for the management of diabetes. This concurs with similar findings reported in the literature (Nasseh et al. 2017).

In Taiwan, the National Health system provides each beneficiary dental scaling every 6 mo. A 13-y retrospective cohort of health claims data included 14,328 adults aged 50 to 64 y (Kao et al. 2021). Those who received scaling more than twice per year were excluded. Participants were matched across all risk variables for acute myocardial infarct (AMI) between those who had regular (i.e., every 6 or 12 mo) scaling and those who did not. The incidence rate of AMI in the group without any dental scaling was 3.5%, significantly higher than the 1.9% in those with regular scaling. The annual costs for tooth scaling plus AMI treatments were approximately USD $35 lower per patient per year than those who had no scaling.

### Risk of Bias in Included Studies

The overall evidence from the included studies in this report is high quality and at low risk of bias (Appendix 2). Three prospective studies (Lee et al. 2019; Smits et al. 2020; Kao et al. 2021) were evaluated using the Newcastle-Ottawa assessment scale for cohort studies. All were judged as low risk of bias. One cluster RCT (Clarkson et al. 2021; Ramsay et al. 2018) demonstrated low risk of bias. Both systematic reviews (Lamont et al. 2018; Manresa et al. 2018) were judged as low risk of bias.

The AGREE II methodology was used to appraise the 2 clinical practice guidelines (Sanz, Herrera, et al. 2020; Herrera et al. 2022). There are 2 limitations to these guidelines. Patients/patient groups were not represented among stakeholders, although there is mention of inclusion of these groups in future updates. It is noted that major changes in relevant evidence will trigger an update and recommendations modified as appropriate, but there is no specific mention of an audit of the implementation of these guidelines. An audit would be a challenging undertaking, and lack thereof is not seen as a major limitation. In all other aspects—rigor of development, clarity of presentation, editorial independence, and applicability—both guidelines are deemed to be of the highest possible quality.

## Discussion

The evidence provided in this review is mixed, with a high level of certainty due to minimal risk of bias in the appraised studies for “regular dental attenders” and those with a diagnosis of periodontal disease and absent for those individuals with low socioeconomic status or living in residential care. Thus, the major limitation of our findings relates to the generalizability of the results to all populations, particularly those eligible for the CDCP.

The patients in the Clarkson et al. (2021), Lamont et al. (2018), Kao et al. (2021), and Lee et al. (2019) studies were “regular dental attenders,” with no periodontal disease or at low risk of developing periodontitis. This group of patients generally has good access to regular dental care and is more likely to have had their oral health maintained throughout most of their lives. However, this does not apply to all Canadians. There is robust evidence that periodontitis disproportionately affects the vulnerable segments of the population and is a significant source of social inequality (Jin et al. 2011; Jepsen et al. 2017). It is clear from data from the Canadian Health Measures Survey (Health Canada 2010) and reports from the Canadian Academy of Health Sciences (2014), for example, that utilization of and access to dental services is unequally distributed. Recent findings from Statistic Canada (StatsCan 2024) state that one-third of those with financial difficulties avoid seeing a dental professional because of costs. Yet, there appears to be no literature evaluating the issues of equity or access to routine preventive periodontal care in this population. Therefore, the robust evidence provided by the aforementioned studies must be applied with caution to those who experience barriers to accessing regular dental care.

The findings from Clarkson (Clarkson et al. 2021; Ramsay et al. 2018) and Lamont et al. (2018) support the controversial recommendation that the current scheduling of routine S&P treatments provides no clinical benefit in terms of control of gingival inflammation—at least over 3 y. This does not seem to be true for the risk of tooth loss, however, the risk of which is accrued over a longer period of time. Ramsey and Lamont also propose that routine S&P may be an inefficient use of scarce resources. An alternative approach could be to redirect funding toward patients with a clear diagnosis of periodontal disease. The Basic Periodontal Exam (BPE) (British Society of Periodontology 2016), for example, is a screening tool to determine gingival and periodontal health. Each score relates to a set of treatment codes eligible for reimbursement. However, unlike the United Kingdom, the Canadian dental infrastructure does not provide a clear process for identifying these patients using diagnostic or risk-based codes.

Evidence presented in this review strongly supports setting the frequency of routine scaling intervals according to an individual patient’s risk status (Sanz, Herrera, et al. 2020; Herrera et al. 2022). Little is known about the implementation of risk-driven recall intervals for supportive periodontal care, and while anecdotal evidence from oral health care professionals suggests that 3-monthly intervals are feasible and acceptable to patients at high risk of recurrence of periodontitis, we do not have adequate data to make specific recommendations for those affected by the Canada Dental Care Program. For this particular population, there are barriers to accessing periodontal treatment and routine periodontal maintenance. Certainly, the aim of the Canada Dental Care Program is to reduce current economic barriers, but unlike the United Kingdom and United States, Canada lacks the infrastructure to report dental diagnostic codes (and thus, in the case of periodontitis, risk profile; Tonetti et al. 2018) to third-party payers. These diagnostic codes would facilitate the determination of appropriateness of care—and dental cost coverage—for an individual.

The focus of this review was on the clinical beneficial effects of S&P for periodontal and gingival health. Untreated dental caries has potential for significant morbidity among older adults and vulnerable children, and there is clear evidence in the literature that regular plaque removal is important to prevent dental caries. However, investigating the impact of routine S&P on dental caries was outside the scope of this report.

There are limitations to this review. Publication bias was not assessed. Broadening the search terms and inclusion criteria may have yielded additional studies to answer the questions for other population groups. Nonetheless, accepted guidelines were followed in conducting a thorough search for the best available research, and a robust analysis of the strength of evidence was conducted. As this was a rapid evidence review rather than a full systematic review with quantitative statistics, a full meta-analysis was not carried out. However, the results are based on a set of clear, well-defined clinical questions from 4 databases. This indicates there may not have been adequate data available in the literature to be able to conduct a statistical analysis.

## Conclusions

We retrieved no literature to support dental polishing alone as an effective means of maintaining gingival and periodontal health. We found no evidence that examined the effects of, or appropriate intervals for, routine scaling in adults with inequitable access to dental care.

In patients diagnosed with periodontitis, once active treatment is complete, there is robust evidence to support routine scaling to reduce risk of tooth loss, prevent periodontal disease recurrence, and, in patients with diabetes mellitus, reduce health care costs. There is strong evidence that scaling intervals of 3 to 12 mo should be tailored to an individual’s risk profile and periodontal conditions.

Based on the available evidence for those with no or early signs of periodontitis who regularly attend general dental offices, there is strong evidence that routine scale and polish may have little or no clinical benefit over 3 y, regardless of the interval. There is a moderate level of certainty that patients who receive scaling once or twice a year are less likely to suffer tooth loss and are at a lower risk of cardiac events, with consequential reduced costs for medical care. Taking into account health and non-health-related costs, there is a moderate level of evidence that scaling and individualized OHI at 6-mo intervals has the largest incremental net benefit and that patients place a high value on routine scaling.

In patients who have traditionally had inequitable access to dental care, such as those eligible for the CDCP, it is well established that this population is at greater risk for periodontitis and more likely to experience the impact of untreated periodontal disease on their overall health than the general population who are “regular dental attenders.” However, in the absence of infrastructure to use diagnostic or risk categories to determine reimbursement for dental services reimbursement for services under a government-sponsored plan such as the CDCP, many people may be “underinsured” and thus undertreated for periodontal disease.

Based on the findings of this review, research on the oral health impacts of the CDCP on this vulnerable population should be prioritized.

## Author Contributions

D. Matthews, contributed to conception and design, data acquisition, analysis, and interpretation, drafted the manuscript; H. Al-Waeli, contributed to data acquisition and interpretation, critically revised the manuscript.

## Supplemental Material

sj-docx-1-jct-10.1177_23800844241271684 – Supplemental material for Benefits of Dental Scaling and Polishing in Adults: A Rapid Review and Evidence Synthesis Supplemental material, sj-docx-1-jct-10.1177_23800844241271684 for Benefits of Dental Scaling and Polishing in Adults: A Rapid Review and Evidence Synthesis by D.C. Matthews and H. Al-Waeli in JDR Clinical & Translational Research
