# Supplementary material for: Benefits of Dental Scaling and Polishing in Adults: A Rapid Review and Evidence Synthesis
Source: JDR Clin Trans Res. 2024 Oct 9;10(3):269–81. doi: 10.1177/23800844241271684 (PMC12166143; doi:10.1177/23800844241271684)
Supplement: sj-docx-1-jct-10.1177_23800844241271684 – Supplemental material for Benefits of Dental Scaling and Polishing in Adults: A Rapid Review and Evidence Synthesis [file sj-docx-1-jct-10.1177_23800844241271684.docx]

**Appendix Table 1: Search Strategy of Databases and Registers**

1. **Cochrane Oral Health Trials Register search**

Search Name: Routine scale - with oral registry code

Date Run: 13/09/2023 12: 23: 46

ID Search Hits

#1 (routine* OR recall* OR regular* OR periodic* OR (six NEXT month*) OR (6 NEXT month*) OR (three NEXT month*) OR (3 NEXT month*)):ti,ab 363785

#2 (scaling OR "scale and polish" OR "dental prophylaxis" OR "oral prophylaxis"):ti,ab 6419

#3 #1 AND #2 2354

#4 SR-ORAL 54100

#5 #3 AND #4 with Cochrane Library publication date Between Jan 2018 and Dec 2023 566

Notes**:** The Cochrane Oral Health Trials Register is not currently being maintained. It is up to date as of June 30, 2022. SR-ORAL code used to search the register.

1. **Cochrane Central Register of Controlled Trials (CENTRAL) search**

Search Name: Routine scale and polish

Date Run: 13/09/2023 12: 31: 55

ID Search Hits

#1 MeSH descriptor: [Periodontal Diseases] explode all trees 8339

#2 (periodonti* OR (periodont* near/3 disease*)):ti,ab,kw 9786

#3 gingivitis:ti,ab,kw 3770

#4 ((gingiva* near/5 inflam*) OR (gingiva* near/5 disease*) OR (gingiva* near/5 bleed*) OR (gingiva* near/5 swell*)):ti,ab,kw 3809

#5 ((gum* near/5 inflam*) OR (gum* near/5 disease*) OR (gum* near/5 bleed*) OR (gum* near/5 swell*)):ti,ab,kw 580

#6 MeSH descriptor: [Dental Plaque] this term only 2850

#7 MeSH descriptor: [Dental Calculus] this term only 253

#8 ((tooth near/5 plaque) OR (teeth near/5 plaque) OR (dental near/5 plaque)):ti,ab,kw 5613

#9 ((tooth near/5 calculus) OR (teeth near/5 calculus) OR (dental near/5 calculus)):ti,ab,kw 421

#10 #1 OR #2 OR #3 OR #4 OR #5 OR #6 OR #7 OR #8 OR #9 18540

#11 MeSH descriptor: [Dental Prophylaxis] explode all trees 1805

#12 ((dental near/5 scal*) OR (tooth near/5 scal*) OR (teeth near/5 scal*) OR (dental near/5 polish*) OR (tooth near/5 polish*) OR (teeth near/5 polish*) OR (dental near/5 prophylax*) OR (tooth near/5 prophylax*) OR (teeth near/5 prophylax*)):ti,ab,kw 3640

#13 (periodont* near/5 scal*):ti,ab,kw 679

#14 #11 OR #12 OR #13 4217

#15 #10 AND #14 with Cochrane Library publication date Between Jan 2018 and Dec 2023 1186

1. **Medline Ovid search**

| **#** | **Query** | **Results from 13 Sep 2023** |
| --- | --- | --- |
| 1 | exp Periodontal Diseases/ | 95,973 |
| 2 | (periodonti$ or (periodont$ adj3 disease$)).ti,ab. | 57,077 |
| 3 | gingivitis.ti,ab. | 8,673 |
| 4 | ((gingiva$ or gum$) adj5 (inflam$ or disease$ or bleed$ or swell$)).ti,ab. | 12,120 |
| 5 | Dental Plaque/ | 17,430 |
| 6 | Dental Calculus/ | 2,780 |
| 7 | ((tooth or teeth or dental) adj5 (plaque or calculus)).ti,ab. | 9,291 |
| 8 | 1 or 2 or 3 or 4 or 5 or 6 or 7 | 132,321 |
| 9 | exp Dental Prophylaxis/ | 8,122 |
| 10 | ((dental or tooth or teeth) and (scal$ or polish$ or prophylax$)).ti,ab. | 21,994 |
| 11 | (periodont$ adj5 scal$).ti,ab. | 924 |
| 12 | 9 or 10 or 11 | 28,493 |
| 13 | 8 and 12 | 8,731 |
| 14 | randomized controlled trial.pt. | 599,789 |
| 15 | controlled clinical trial.pt. | 95,422 |
| 16 | randomized.ab. | 616,745 |
| 17 | placebo.ab. | 241,407 |
| 18 | drug therapy.fs. | 2,622,278 |
| 19 | randomly.ab. | 416,452 |
| 20 | trial.ab. | 663,657 |
| 21 | groups.ab. | 2,568,280 |
| 22 | 14 or 15 or 16 or 17 or 18 or 19 or 20 or 21 | 5,752,051 |
| 23 | exp animals/ not humans.sh. | 5,154,701 |
| 24 | 22 not 23 | 5,022,748 |
| 25 | 13 and 24 | 4,004 |
| 26 | (2018* or 2019* or 2020* or 2021* or 2022* or 2023*).dt,ez,ed. | 9,167,949 |
| 27 | 25 and 26 | 1,124 |

1. **Embase Elsevier search**

| Id. | Query | Results | Date |
| --- | --- | --- | --- |
| #34. | (('periodontal disease'/exp OR (periodonti*:ti,ab   OR ((periodont* NEAR/3 disease*):ti,ab)) OR  gingivitis:ti,ab OR ((gingiva* OR gum*) NEAR/5  (inflam* OR disease* OR bleed* OR swell*)):ti,ab  OR 'tooth plaque'/de OR 'tooth calculus'/de OR  ((tooth OR teeth OR dental) NEAR/5 (plaque OR  calculus)):ti,ab) AND (((dental:ti,ab OR  tooth:ti,ab OR teeth:ti,ab) AND (scal*:ti,ab OR  polish*:ti,ab OR prophylax*:ti,ab)) OR  (periodont* NEAR/5 scal*):ti,ab)) AND  (('randomized controlled trial'/de OR 'controlled  clinical trial'/de OR random*:ti,ab OR  'randomization'/de OR 'intermethod comparison'/de  OR placebo:ti,ab OR (compare:ti OR compared:ti OR  comparison:ti) OR ((evaluated:ab OR evaluate:ab  OR evaluating:ab OR assessed:ab OR assess:ab) AND  (compare:ab OR compared:ab OR comparing:ab OR  comparison:ab)) OR (open NEXT/1 label):ti,ab OR  ((double OR single OR doubly OR singly) NEXT/1  (blind OR blinded OR blindly)):ti,ab OR 'double  blind procedure'/de OR 'parallel group$':ti,ab OR  (crossover:ti,ab OR 'cross over':ti,ab) OR  ((assign* OR match OR matched OR allocation)  NEAR/5 (alternate OR group$ OR intervention$ OR  patient$ OR subject$ OR participant$)):ti,ab OR  (assigned:ti,ab OR allocated:ti,ab) OR  (controlled NEAR/7 (study OR design OR  trial)):ti,ab OR (volunteer:ti,ab OR  volunteers:ti,ab) OR trial:ti) NOT (('animal'/exp  OR animal:de OR 'nonhuman'/de) NOT ('human'/exp  OR 'human cell'/de OR human:ti OR humans:ti)))  AND [11-01-2018]/sd | 881 | 13 Sep 2023 |
| #33. | ('randomized controlled trial'/de OR 'controlled   clinical trial'/de OR random*:ti,ab OR  'randomization'/de OR 'intermethod comparison'/de  OR placebo:ti,ab OR (compare:ti OR compared:ti OR  comparison:ti) OR ((evaluated:ab OR evaluate:ab  OR evaluating:ab OR assessed:ab OR assess:ab) AND  (compare:ab OR compared:ab OR comparing:ab OR  comparison:ab)) OR (open NEXT/1 label):ti,ab OR  ((double OR single OR doubly OR singly) NEXT/1  (blind OR blinded OR blindly)):ti,ab OR 'double  blind procedure'/de OR 'parallel group$':ti,ab OR  (crossover:ti,ab OR 'cross over':ti,ab) OR  ((assign* OR match OR matched OR allocation)  NEAR/5 (alternate OR group$ OR intervention$ OR  patient$ OR subject$ OR participant$)):ti,ab OR  (assigned:ti,ab OR allocated:ti,ab) OR  (controlled NEAR/7 (study OR design OR  trial)):ti,ab OR (volunteer:ti,ab OR  volunteers:ti,ab) OR trial:ti) NOT (('animal'/exp  OR animal:de OR 'nonhuman'/de) NOT ('human'/exp  OR 'human cell'/de OR human:ti OR humans:ti)) | 5,122,437 | 13 Sep 2023 |
| #32. | ('animal'/exp OR animal:de OR 'nonhuman'/de) NOT   ('human'/exp OR 'human cell'/de OR human:ti OR  humans:ti) | 7,758,182 | 13 Sep 2023 |
| #31. | 'randomized controlled trial'/de OR 'controlled   clinical trial'/de OR random*:ti,ab OR  'randomization'/de OR 'intermethod comparison'/de  OR placebo:ti,ab OR (compare:ti OR compared:ti OR  comparison:ti) OR ((evaluated:ab OR evaluate:ab  OR evaluating:ab OR assessed:ab OR assess:ab) AND  (compare:ab OR compared:ab OR comparing:ab OR  comparison:ab)) OR (open NEXT/1 label):ti,ab OR  ((double OR single OR doubly OR singly) NEXT/1  (blind OR blinded OR blindly)):ti,ab OR 'double  blind procedure'/de OR 'parallel group$':ti,ab OR  (crossover:ti,ab OR 'cross over':ti,ab) OR  ((assign* OR match OR matched OR allocation)  NEAR/5 (alternate OR group$ OR intervention$ OR  patient$ OR subject$ OR participant$)):ti,ab OR  (assigned:ti,ab OR allocated:ti,ab) OR  (controlled NEAR/7 (study OR design OR  trial)):ti,ab OR (volunteer:ti,ab OR  volunteers:ti,ab) OR trial:ti | 5,884,642 | 13 Sep 2023 |
| #30. | trial:ti | 406,016 | 13 Sep 2023 |
| #29. | volunteer:ti,ab OR volunteers:ti,ab | 283,446 | 13 Sep 2023 |
| #28. | (controlled NEAR/7 (study OR design OR   trial)):ti,ab | 450,612 | 13 Sep 2023 |
| #27. | assigned:ti,ab OR allocated:ti,ab | 488,520 | 13 Sep 2023 |
| #26. | ((assign* OR match OR matched OR allocation)   NEAR/5 (alternate OR group$ OR intervention$ OR  patient$ OR subject$ OR participant$)):ti,a | 412,824 | 13 Sep 2023 |
| #25. | crossover:ti,ab OR 'cross over':ti,ab | 124,529 | 13 Sep 2023 |
| #24. | 'parallel group$':ti,ab | 31,990 | 13 Sep 2023 |
| #23. | 'double blind procedure'/de | 210,502 | 13 Sep 2023 |
| #22. | ((double OR single OR doubly OR singly) NEXT/1   (blind OR blinded OR blindly)):ti,ab | 275,319 | 13 Sep 2023 |
| #21. | (open NEXT/1 label):ti,ab | 108,320 | 13 Sep 2023 |
| #20. | (evaluated:ab OR evaluate:ab OR evaluating:ab OR   assessed:ab OR assess:ab) AND (compare:ab OR  compared:ab OR comparing:ab OR comparison:ab | 2,765,414 | 13 Sep 2023 |
| #19. | compare:ti OR compared:ti OR comparison:ti | 626,220 | 13 Sep 2023 |
| #18. | placebo:ti,ab | 364,966 | 13 Sep 2023 |
| #17. | 'intermethod comparison'/de | 301,650 | 13 Sep 2023 |
| #16. | 'randomization'/de | 98,156 | 13 Sep 2023 |
| #15. | random*:ti,ab | 1,965,924 | 13 Sep 2023 |
| #14. | 'controlled clinical trial'/de | 440,671 | 13 Sep 2023 |
| #13. | 'randomized controlled trial'/de | 782,382 | 13 Sep 2023 |
| #12. | ('periodontal disease'/exp OR (periodonti*:ti,ab   OR ((periodont* NEAR/3 disease*):ti,ab)) OR  gingivitis:ti,ab OR ((gingiva* OR gum*) NEAR/5  (inflam* OR disease* OR bleed* OR swell*)):ti,ab  OR 'tooth plaque'/de OR 'tooth calculus'/de OR  ((tooth OR teeth OR dental) NEAR/5 (plaque OR  calculus)):ti,ab) AND (((dental:ti,ab OR  tooth:ti,ab OR teeth:ti,ab) AND (scal*:ti,ab OR  polish*:ti,ab OR prophylax*:ti,ab)) OR  (periodont* NEAR/5 scal*):ti,ab) | 5,272 | 13 Sep 2023 |
| #11. | ((dental:ti,ab OR tooth:ti,ab OR teeth:ti,ab) AND   (scal*:ti,ab OR polish*:ti,ab OR  prophylax*:ti,ab)) OR (periodont* NEAR/5  scal*):ti,ab | 25,042 | 13 Sep 2023 |
| #10. | (periodont* NEAR/5 scal*):ti,ab | 890 | 13 Sep 2023 |
| #9. | (dental:ti,ab OR tooth:ti,ab OR teeth:ti,ab) AND   (scal*:ti,ab OR polish*:ti,ab OR  prophylax*:ti,ab) | 24,563 | 13 Sep 2023 |
| #8. | 'periodontal disease'/exp OR (periodonti*:ti,ab   OR ((periodont* NEAR/3 disease*):ti,ab)) OR  gingivitis:ti,ab OR ((gingiva* OR gum*) NEAR/5  (inflam* OR disease* OR bleed* OR swell*)):ti,ab  OR 'tooth plaque'/de OR 'tooth calculus'/de OR  ((tooth OR teeth OR dental) NEAR/5 (plaque OR  calculus)):ti,ab | 155,943 | 13 Sep 2023 |
| #7. | ((tooth OR teeth OR dental) NEAR/5 (plaque OR   calculus)):ti,a | 9,960 | 13 Sep 2023 |
| #6. | 'tooth calculus'/de | 3,653 | 13 Sep 2023 |
| #5. | 'tooth plaque'/de | 22,597 | 13 Sep 2023 |
| #4. | ((gingiva* OR gum*) NEAR/5 (inflam* OR disease*   OR bleed* OR swell*)):ti,ab | 13,912 | 13 Sep 2023 |
| #3. | gingivitis:ti,ab | 9,495 | 13 Sep 2023 |
| #2. | periodonti*:ti,ab OR ((periodont* NEAR/3   disease*):ti,ab) | 60,230 | 13 Sep 2023 |
| #1. | 'periodontal disease'/exp | 126,705 | 13 Sep 2023 |

Notes: Ovid wildcard ($) means unlimited right-hand truncation, where it doesn’t in Embase through Elsevier, so $ changed to truncation (*) to translate this function. ADJ with no number finds words in the order they are entered with no words in between, so translated to NEXT/1 in Embase Elsevier to capture this function. $1 not available in Elsevier, but $ has the same function, so changed from $1 to $. Translated field code .hw. to :de. Added quotation marks to search phrases.

**Appendix Table 2: Summary of Risk of Bias Assessment of Included Studies**

| **Newcastle Ottawa Scale (NOS) for cohort studies** (2021) **^¶^** | | | | |
| --- | --- | --- | --- | --- |
| *Domains* | **Smits et al.** (2020) | **Lee et al.** (2019) | | **Kao et al.** (2021) |
| Selection of cohort | ★(low) | ★ (low) | | ★★ (low) |
| Comparability of controls | ★ (low) | ★ (low) | | ★★ (low) |
| Outcome assessment | ★ (low) | ★low) | | ★★ (low) |
| **Cochrane Risk of Bias 2 for randomized trials** (2021) | | | | |
| *Domains* | **Clarkson et al.** (2021) | | | |
| Randomization | Low | | | |
| Deviations from intended interventions | Low | | | |
| Missing outcome data | Low | | | |
| Outcome measurement | Low | | | |
| Reporting results | Low | | | |
| Overall risk of bias | Low | | | |
| **ROBIS (Systematic Reviews) (2023)** | | | | |
| *Domains* | **Lamont et al**. (2018) | | **Manresa et al.** (2018) | |
| Study eligibility criteria | Low | | Low | |
| Identification and selection of studies | Low | | Low | |
| Data collection and study appraisal | Low | | Low | |
| Synthesis of findings | Low | | Low | |
| Risk of bias of review | Low | | Low | |
| **AGREE II (Guidelines)** (Brouwers et al. 2010**)*** | | | | |
| *Domains* | **Sanz et al.** (2020b) | | **Herrera et al.** (2022) | |
| Scope and purpose | 7 | | 7 | |
| Stakeholder involvement** | 5 | | 5 | |
| Rigor of development | 7 | | 7 | |
| Clarity of presentation | 7 | | 7 | |
| Applicability** | 5 | | 5 | |
| Editorial independence | 7 | | 7 | |
| Overall Guideline Assessment | 7 | | 7 | |

¶ A study can be awarded a maximum of one star (representing low risk) for each within the Selection and Outcome categories. A maximum of two stars can be given for Comparability.

*AGREE Rating scale 1-7, 7 being least risk of bias

**Sanz and Herrera. For both guidelines, patients/patient groups were not represented among stakeholders, although there is mention of inclusion of these groups in future updates. There was no specific mention of audit of the implementation of these guidelines.
